# Combination Therapy of Navitoclax with Chemotherapeutic Agents in Solid Tumors and Blood Cancer: A Review of Current Evidence

**DOI:** 10.3390/pharmaceutics13091353

**Published:** 2021-08-28

**Authors:** Nur Syahidah Nor Hisam, Azizah Ugusman, Nor Fadilah Rajab, Mohd Faizal Ahmad, Michael Fenech, Sze Ling Liew, Nur Najmi Mohamad Anuar

**Affiliations:** 1 Programme of Biomedical Science, Centre for Toxicology & Health Risk Studies, Faculty of Health Sciences, Universiti Kebangsaan Malaysia, Jalan Raja Muda Abdul Aziz, Kuala Lumpur 50300, Malaysia; p104164@siswa.ukm.edu.my (N.S.N.H.); sl.liew18@gmail.com (S.L.L.); 2 Department of Physiology, Faculty of Medicine, Universiti Kebangsaan Malaysia Medical Centre, Jalan Yaacob Latif, Bandar Tun Razak, Cheras, Kuala Lumpur 56000, Malaysia; dr.azizah@ppukm.edu.my; 3 Center for Healthy Ageing & Wellness, Programme of Biomedical Science, Faculty of Health Sciences, Universiti Kebangsaan Malaysia, Jalan Raja Muda Abdul Aziz, Kuala Lumpur 50300, Malaysia; nfadilah@ukm.edu.my (N.F.R.); michael.fenech@unisa.edu.au (M.F.); 4 Department of Obstetrics and Gynaecology, Faculty of Medicine, Universiti Kebangsaan Malaysia Medical Centre, Jalan Yaacob Latif, Bandar Tun Razak, Cheras, Kuala Lumpur 56000, Malaysia; drmohdfaizal@ukm.edu.my; 5 Genome Health Foundation, North Brighton, SA 5048, Australia

**Keywords:** navitoclax (ABT-263), anti-cancer, combination regimen, solid tumor, non-solid tumor

## Abstract

Combination therapy emerges as a fundamental scheme in cancer. Many targeted therapeutic agents are developed to be used with chemotherapy or radiation therapy to enhance drug efficacy and reduce toxicity effects. ABT-263, known as navitoclax, mimics the BH3-only proteins of the BCL-2 family and has a high affinity towards pro-survival BCL-2 family proteins (i.e., BCL-XL, BCL-2, BCL-W) to induce cell apoptosis effectively. A single navitoclax action potently ameliorates several tumor progressions, including blood and bone marrow cancer, as well as small cell lung carcinoma. Not only that, but navitoclax alone also therapeutically affects fibrotic disease. Nevertheless, outcomes from the clinical trial of a single navitoclax agent in patients with advanced and relapsed small cell lung cancer demonstrated a limited anti-cancer activity. This brings accumulating evidence of navitoclax to be used concomitantly with other chemotherapeutic agents in several solid and non-solid tumors that are therapeutically benefiting from navitoclax treatment in preclinical studies. Initially, we justify the anti-cancer role of navitoclax in combination therapy. Then, we evaluate the current evidence of navitoclax in combination with the chemotherapeutic agents comprehensively to indicate the primary regulator of this combination strategy in order to produce a therapeutic effect.

## 1. Introduction

An early review has described six mechanistic strategies of cancer cells to dictate malignant growth [1,2]. Later, a recent study has re-evaluated and reported more updated cancer hallmarks consisting of seven factors; (i) selective proliferative advantage; (ii) altered stress response; (iii) vessel development; (iv) invasion and metastasis; (v) metabolic reconfiguration; (vi) immune regulation; and (vii) an abetting micro-environment [3]. The first hallmark was explained by the collaboration between oncogenes’ activation with tumor suppressor genes’ inactivation at the cellular level. Next, the second factor was modified from the Hanahan & Weinberg study, in which they quoted as evading programmed cell death and unlimited proliferative capability. However, cancer cells are going through cellular senescence and apoptotic events as well [4]. Such events would increase the selective pressure on cancer cells, and those cells that can adapt better to the situation would survive [5]. In addition, the apoptosis of (pre)-cancerous cells would allow repopulation by more aggressive tumor cells, potentially driving tumor evolution [6]. Emerging studies reported both angiogenesis and vascularization discretely modulate the initiation of microtumors [3,7]. Angiogenesis influences exponential tumor growth, whilst vascularization is vital for cell survival and spreading [8]. Malignant tumor metastasis involves the invasion of adjacent tissues, and this event is often responsible for more than 90% of cancer-related deaths [9]. The discovery of metabolic alterations in cancer cells provides novel insight into the causes and consequences of this factor towards the initiation and tumor progression. Several metabolic alterations are associated with cancer, including the elevation of nitrogen demand, deregulated uptake of nutrients such as glucose and amino acids, and changes in metabolite-driven gene regulation [10,11]. Besides, several pre-existing pathological conditions, such as chronic inflammatory, hyperglycemic, hypoxia, and glycoxidative stress, in conjunction with the activation of the receptor for advanced glycation end products (RAGE)-ligand would synergistically promote tumor development and progression, mostly in diabetic and obese patients [12]. Immune regulation in cancer is postulated to play a prominent role during the initiation and progression of tumorigenesis. Lastly, an abetting and dynamic microenvironment is produced through a continuous paracrine interaction between cancerous and stromal cells at all stages of carcinogenesis, resulting in tumor progression and survival of the cancer cells [3]. 

As mentioned above, resistance to cellular apoptosis is one of the cancer hallmark domains as it causes an excessive, uncontrolled proliferation of cancer cells and promotes tumor metastasis. Anti-apoptotic BCL-2 family proteins largely contribute to the survival of cancer cells, as many studies demonstrated the upregulation of these proteins involved in cancer progression and resistance to chemotherapy treatment [13,14,15]. This finding is comparable with the report from The Human Protein Atlas database, where different expression levels of the anti-apoptotic BCL-2 genes and proteins were detected in solid tumors and lymphoid malignancies [16,17]. The BCL-2 family protein is classified into three groups according to its functions and structures. The first group consists of multi-BH domain pro-apoptotic proteins (BAX and BAK), which act as apoptosis effectors; the second group includes the anti-apoptotic proteins (BCL-2, BCL-XL, BCL-W, MCL-1 and BFL-1), which prevent cell apoptosis; and the third group, which is comprised of BH3-only pro-apoptotic proteins (Noxa, Bad, Bim and Puma), can initiate cell apoptosis and counteract certain anti-apoptotic proteins [13,18]. The interaction among the BCL-2 family protein groups is complex. It is characterized by a direct and indirect signaling activation upon receiving a trigger due to cell death together with DNA damage signals. The signal from their interactions can stimulate and also sensitize BH3-only activator proteins. The activation of these proteins triggers the mitochondrial outer membrane permeabilization (MOMP) through the oligomerization of multidomain pro-apoptotic proteins. The stimulation of MOMP leads to cytochrome c release, caspase activation, and eventually apoptosis. However, this can be blocked by multidomain, anti-apoptotic BCL-2 family proteins. BH3-only sensitizer proteins can reverse this inhibition and indirectly induce apoptosis by binding to the multidomain, anti-apoptotic proteins, releasing the BH3-only activator proteins from the anti-apoptotic proteins [19]. 

**Figure 1:**
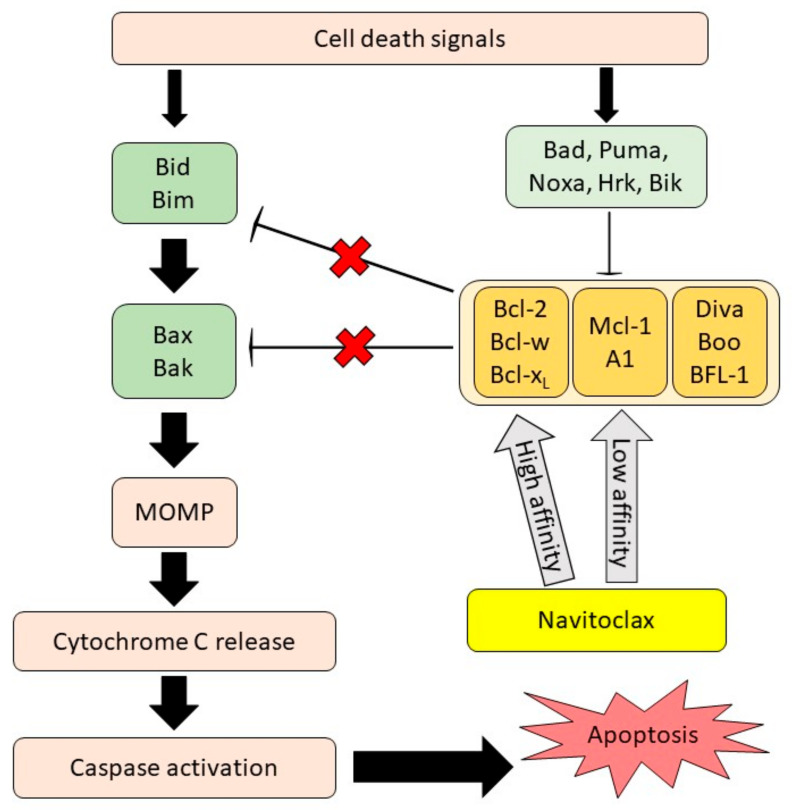
**Navitoclax mechanism of action.** Navitoclax potentiates the intrinsic cell death mechanism through the inhibition of anti-apoptotic proteins signal. Navitoclax affinity on anti-apoptotic proteins is varied. MOMP, mitochondrial outer membrane permeabilization, reproduced from [26], Frontiers, 2020.

The development of various BCL-2 inhibitors as the tumor cells’ apoptosis regulators is evolving as a single drug or administered with other therapeutic agents. Some of them have been implemented in human clinical trials [20] and have been approved by the U.S. Food and Drug Administration (FDA) [21,22]. In 2008, a small molecule BH3-mimetic drug called navitoclax was developed as an analogue to ABT-737 and displayed better oral bioavailability than its predecessor [23]. Navitoclax has been widely used in clinical studies for cancer treatment due to its nature as a selective inhibitor of the BCL-2, BCL-XL and BCL-W proteins [23]. It can mimic the function of the BH3-only proteins and bind to the anti-apoptotic BCL-2 proteins, thus allowing the intrinsic apoptosis mechanism activation [24]. The anti-cancer effect of navitoclax mainly relies on the blocking of the BCL-2 family members, as shown in Figure 1. When navitoclax binds to BCL2, BCL-XL or BCL-W, the effectors of apoptosis, namely BAX and BAK, will be released from the BCL-2 proteins to carry out their functions. BAX and BAK will then oligomerize at the outer membrane of the mitochondria and activate caspase, thereby inducing apoptosis [25].

The mechanism of navitoclax in enhancing cancer cell death is mainly dependent on the mitochondrial intrinsic apoptosis pathway. Navitoclax exhibits significant single-agent efficacy against cancer cells with an overexpression of BCL-2 or BCL-XL proteins [27] and yields synergistic effects with other drugs in various diseases [28]. In a previous review, we presented and discussed the ability of navitoclax to mediate pro-apoptotic and anti-fibrotic action as a single agent in various cancer types [26]. However, the combination therapy of navitoclax is not highlighted and evaluated thoroughly, in light of the fact that the utilization of navitoclax with other chemotherapeutic agents has demonstrated promising, therapeutic outcomes in several solid and non-solid tumor clinical studies. Therefore, this manuscript aims to report the clinical evidence of navitoclax combination therapy meticulously, evaluate the clinical studies’ results and limitations, and provide a proposed future direction of this drug development.

## 2. The Rationale for Navitoclax Combination Therapy in Solid and Non-Solid Tumors Treatment

Combining two or more anti-cancer agents is actively evolving, which fundamentally aims to reduce the chance of cancer cells becoming resistant to these therapeutic agents and hindering cancer recurrence. This approach was started in the early 1960s19 and 1970s19, whereby scientists sought a way to combat refractory and relapse cancer [29]. A commentary reported the utilization of nitrogen mustard, Oncovin, methotrexate and prednisone concomitantly to treat Hodgkin’s lymphoma, and it showed promising outcomes with manageable toxicity effects [30,31]. Next, MOPP therapy was introduced involving nitrogen mustard, Oncovin, procarbazine and prednisone as an upgraded version of MOMP with the ultimate treatment protocol. The results showed that patients with very advanced Hodgkin’s disease attained a complete remission characterized by having an utmost tumor suppression and prolonged survival rate, with the absence of cancer recurrence after 40 years [32]. A single chemotherapy drug may be effective against some types of cancer [33,34]. However, doctors often give several chemotherapy drugs simultaneously due to the complex cancer pathophysiology, and thereby multiple modulators are required to fight the cancer cell and tumor metastasis [35]. Apart from that, different types of cancer may act heterogeneously to the cancer treatment options, such as in “responsive cancers”, which includes lymphomas and breast cancers, which manage to respond well to radiation therapy or chemotherapy [34,36,37], whilst people with so-called “resistant cancers”, such as malignant brain tumors or skin cancers, may not effectively respond to the chemotherapy or radiation therapy [38,39]. Some intestinal tract or lung tumors are often responding initially to chemotherapy, but later become resistant despite continued treatment [40,41]. Different reactions of different tumor types towards cancer-targeted therapy have provided an impetus towards a new strategy that utilizes anti-cancer drugs alone or with other chemotherapeutic drugs. Occasionally, combination drug therapy is used not to cure, but to minimize the symptoms, treat the side effects of chemotherapeutic agents, and prolong survival rates [42]. Besides, the utilization of multiple anti-cancer agents provides advanced therapeutic outcomes simultaneously, including tumor growth and metastatic potential reduction, triggering programmed cell death, lessening the population of cancer stem cells, and arresting mitotically active cells [43]. To date, both chemotherapy and targeted therapy have significantly improved the survival and quality of life of cancer patients and sometimes induce a complete tumor remission [29]. Classical chemotherapy exhibits a potent cytotoxicity effect on cancer cells and healthy cells since they are known as cytostatic drugs that often can induce severe adverse effects [44]. Meanwhile, the development of targeted therapy is fundamentally designed to specifically act on cancer cells, targeted genes, or proteins that involve tumor growth and progression [45]. Not only that, targeted therapy has been demonstrated to ameliorate chemotherapy and minimize the severe adverse events induced by chemotherapy. Due to that, a combination therapy strategy has shown promising results in alleviating the burden of various tumor types [46,47]. 

Strategy for targeting BCL-2-associated pathways in cancer includes developing BH3 mimetics to promote apoptosis [48,49]. These BH3 mimetics act as inhibitors against anti-apoptotic proteins by blocking the BCL-2 and BAK/BAX interaction [26,48]. In the past, a small molecular inhibitor, called gossypol, had the most successful anti-cancer effects in clinical trials [50]. It was shown to be efficacious against various tumor types, such as breast, prostate, and non-small cell lung cancer (NSCLC). Unfortunately, this benefit did not extend to phase II clinical trials owing to the low binding affinity and toxic side effects [51]. The further development of BH3 mimetics gave rise to the discovery of navitoclax [23]. Navitoclax exhibited promising results as a single agent preclinically against various solid tumors, such as small cell lung cancer (SCLC) [52], epithelial cancer [53], breast cancer [54], and oral cancer [55], as well as non-solid tumors, including hematologic tumors [28] and lymphoid malignancies [23]. Phase I clinical trials of navitoclax against SCLC [56] and chronic lung cancer [25] demonstrated a relatively favorable outcome, though most patients experienced dose-dependent thrombocytopenia. Meanwhile, phase II studies in patients with recurrent and progressive SCLC had demonstrated a restricted, single-agent activity of navitoclax [57]. This result is contrary to the previous work on the SCLC xenograft model of mice, which showed significant tumor cell apoptosis and suppressed tumor in the xenograft model [52]. However, BCL-2 and MCL-1 were identified as critical resistance factors to chemotherapy that enhanced SCLC survival. Since navitoclax exhibited an anti-apoptotic effect by inhibiting BCL-XL activity in SCLC in an in vitro study [23,26], hence, the utilization of navitoclax in combination with other anti-cancer agents should be implemented to target these anti-apoptotic proteins (i.e., BCL-XL, BCL-2, MCL-1) at once, thereby achieving the synergistic anti-cancer effect. Our previous review has elucidated that the navitoclax toxicity effect would involve reducing circulating platelet counts in the blood, attributed to the blocking of the BCL-XL activity [26]. Other than that, the efficacy of navitoclax on particular cancer types, such as acute lymphocytic leukemia (ALL) and advanced SCLC, is limited due to different expression levels of the BCL-2 family protein, which can influence the navitoclax’s therapeutic effect. 

## 3. Clinical Studies of Navitoclax Combination Therapy

Many studies have been conducted on the combination of navitoclax with other BCL-2 family inhibitors or other chemotherapeutic agents to examine their effects against different solid and non-solid malignancies. The combined treatment of the drugs was shown to stimulate anti-cancer activities through the synergistic interactions and inhibition of certain anti-apoptotic proteins to improve the efficacy of navitoclax and minimize its potential side effects. The clinical studies and outcomes of navitoclax in combination therapy are summarized in Table 1.

### 3.1. Solid Tumors

#### 3.1.1. Metastatic Melanoma

The incidence of metastatic melanoma has increased because of the incomplete response to treatment or relapse from treating a subset of melanomas [39]. The anti-apoptotic BCL-2 proteins, including MCL-1 and BCL-XL, play a critical role in the survival of these hard-to-treat melanoma cells. Mukherjee et al. (2020) observed that the genetic knockdown of BCL-XL by short hairpin RNA (shRNA) stimulated the responsiveness of melanoma towards the MCL-1 blocker S63845. In contrast, the genetic knockdown of MCL-1 promoted the responsiveness of melanoma towards the BCL-XL inhibitor navitoclax. A combination of the MCL-1 blocker S63845 and the BCL-XL blocker navitoclax acted synergistically to attenuate different types of melanoma cells in vitro and in vivo [58]. This two-drug combination exhibited a higher significant effect as compared to the application of a single drug. By targeting the anti-apoptotic modulators (BCL-XL and MCL-1) of melanoma, this combination could provide a new therapeutic efficacy against metastatic melanoma resistant to ongoing treatment [58].

#### 3.1.2. Rhabdomyosarcoma (RMS)

One of the main factors contributing to metastasis, cancer cell survival, and resistance to treatment in alveolar rhabdomyosarcoma (RMS) is the upregulation of the oncogenic fusion protein PAX3-FOXO1 [59]. It was found that the application of a single-drug treatment against RMS increased the tendency of the rhabdomyosarcoma cells to be more drug resistant. Based on a study conducted by Ommer et al. (2020), the activity of caspase 3/7 in fusion-positive (F.P.) RMS cell lines and patient-derived xenograft (PDX) increased significantly following the loss of the PAX3-FOXO1 protein induced by alisertib, an aurora kinase A (AURKA) inhibitor. These RMS cells then carried out intrinsic apoptosis mediated by the BH-3 only pro-apoptotic Noxa protein. Significantly, apoptosis of the RMS cells could be augmented by the BH3-mimetic navitoclax after the loss of PAX3-FOXO1. Hence, the combination treatment of navitoclax and alisertib could produce a synergistic effect against RMS cell lines in vitro and inhibit RMS tumor progression in vivo [60]. 

#### 3.1.3. Endometrial Carcinoma

Endometrial carcinoma is a malignant epithelial tumor that occurs in the endometrium with high incidence and mortality [61]. To date, surgery is still the first choice of treatment for endometrial carcinoma. However, the probability of relapse after the operation is still high, requiring chemotherapy application [62]. Combined therapy with cisplatin, paclitaxel, and doxorubicin (DOX) faces a significant challenge in terms of the poor drug distribution to cancer cells, difficulty in achieving the synergistic outcome, and severe side effects [63]. Moreover, the effect of DOX is strongly inhibited by the overexpression of the anti-apoptotic BCL-2 protein. In order to deliver multiple drugs to cancer cells simultaneously, Ding et al. (2020) generated an ultra pH-sensitive nanoparticle based on polyethylene glycol-poly(diisopropylamino)ethyl methacrylate (PEG-PDPA), with doxorubicin encapsulated in the hydrophilic cavity and a BCL-2 inhibitor navitoclax encapsulated in the hydrophobic membrane. Upon arriving at the tumor site, the accumulated nanoparticles were endocytosed into the cancer cells. The acidic pH in lysosomes would then trigger the release of DOX and navitoclax immediately. Hence, this combination therapy between DOX and navitoclax significantly enhanced the endometrial tumor-killing effect in vivo and in vitro [64].

#### 3.1.4. Papillary Thyroid Cancer (PTC)

The BRAFV600E mutation in papillary thyroid cancer (PTC) is often associated with aggressive tumor characteristics, such as metastasis, cancer recurrence, and the failure of radioiodine treatment [65]. Vemurafenib, a potent BRAF inhibitor that demonstrated strong efficiency in metastatic malignant melanoma with the BRAFV600E mutation [66] also showed a good therapeutic effect against BRAFV600E-positive PTC [38,67]. However, the development of resistance towards vemurafenib slowly restricted its efficacy in BRAFV600E-positive PTC [68]. According to Jeong et al. (2019), vemurafenib alone exhibited an anti-proliferative effect against K1 BRAFV600E-positive PTC cells by suppressing almost half of the K1 cells’ growth at 10 µM. After the treatment of vemurafenib for 24 h, the protein expression of p-Erk1/2 reduced, while the protein expression of BCL-XL and BCL-2 increased. The data proved that vemurafenib increased the expression of anti-apoptotic BCL-2 and BCL-XL protein in K1 cells. On the other hand, 24 h treatment of 4 µM of navitoclax alone showed insignificant outcomes on the survival of the K1 cells. Therefore, a combination of navitoclax and vemurafenib was given against K1 BRAFV600E-positive PTC cells. This combination significantly inhibited cell development and induced a higher apoptosis rate with a lower concentration of navitoclax and vemurafenib, in which 0.5 µM and 1 µM were required respectively to yield synergistic activity [69].

#### 3.1.5. Pancreatic Ductal Adenocarcinoma (PDAC)

Pancreatic ductal adenocarcinoma (PDAC) is the fatal form of pancreatic cancer and contributes to a high number of deaths among pancreatic cancer patients [70]. The poor clinical prognosis is often due to limited treatment alternatives. An overexpression of the anti-apoptotic BCL-XL and MCL-1 protein in PDAC helps cancer cells to avoid apoptosis. Thus, targeting both BCL-XL and MCL-1 provides a new treatment option for PDAC [71,72]. Nevertheless, navitoclax as a BCL-XL inhibitor was not sufficient to work as a single agent against PDAC due to the resistance of the anti-apoptotic MCL-1 protein towards navitoclax [73]. Subsequently, the inhibition of MCL-1 was required to sensitize cancer cells to navitoclax [74]. Cyclin-dependent kinase 5 (CDK5) is known to regulate the stability of MCL-1 [74], and the inhibition of CDK5 will lead to the breakdown of MCL-1 [75]. Kour et al. (2019) studied the effect of aminopyrazole on CDK5 and proved that aminopyrazole analogue 24 could inhibit CDK5 kinase activity. Apoptosis of pancreatic cancer cell lines could only be achieved by combining analogue 24 and navitoclax. This showed that the simultaneous inhibition of BCL-XL and MCL-1 is essential to triggering apoptosis in pancreatic cancer cell lines. The increased apoptosis could be observed in the increase of caspase 3/7. In short, the combined treatment of the CDK5 inhibitor analogue 24 and the BCL-XL inhibitor navitoclax synergistically stimulated apoptosis and suppressed cancer cell growth in pancreatic cancer cell lines compared to single-drug therapy [76]. 

#### 3.1.6. Small Cell Lung Carcinoma (SCLC)

SCLC is a destructive type of cancer with increasing patient mortality risk. Over the past few decades, no appropriate treatment has been found effective against SCLC [41,77]. The survival and resistance of SCLC cells are mainly attributed to the overexpression of anti-apoptotic BCL-2 family members, such as BCL-XL and MCL-1. Hence, studies hypothesized that blocking the anti-apoptotic proteins could overcome the therapeutic resistance. BCL-2 inhibitor navitoclax can bind to both BCL-2 and BCL-XL with high affinity but exhibit low affinity towards MCL-1 protein. In other words, navitoclax does not inhibit MCL-1 activity [23]. The presence of BH3-only pro-apoptotic Noxa proteins is known to increase the sensitivity of SCLC cells towards navitoclax. This can be achieved when Noxa binds to MCL-1 from the cytoplasm to the mitochondria, contributing to MCL-1 phosphorylation and subsequent degradation [46]. Recently, Noxa has been proven to be upregulated by histone deacetylase (HDAC) inhibitors [78]. Thus, Nakajima et al. (2016) combined vorinostat (an HDAC inhibitor) with navitoclax (a BCL-XL inhibitor) to treat SCLC cell lines. The combination of vorinostat and navitoclax significantly enhanced apoptosis in SCLC cell lines, including navitoclax-resistant cells. SCLC cell death activated by this combination is Noxa-dependent in certain cell lines, while in others it is due to the blocking of BCL-XL and subsequent release of BAK from BCL-XL and MCL-1 [46].

### 3.2. Non-Solid Tumors

#### 3.2.1. Acute Myeloid Leukemia (AML)

Early studies discovered the occurrence of nucleoporin 98kD/nuclear receptor binding SET Domain Protein 1 (NUP98/NSD1) fusion as a result of chromosomal translocation at t(5;11)(g35;p15.5) gene in both pediatric and adult positive AML patients [79]. NUP98/NSD1-positive AML patients exhibit deleterious phenotypes indicated by the higher leukocytes counts and more myelomonocytic leukemia/monocytic leukemia classification (FAB-M4/M5) morphology [79]. Further research reported the majority of NUP98/NSD1-positive patients had internal tandem duplications (ITD) in the cytoplasmic domain of the FLT3 gene that causes the activation of constitutive kinase (FLT3-UT3 mutation) [80]. The coexistence of the NUP98-NSD1 and the FLT3-UT3 mutation in leukemic cells exhibited a high sensitivity towards the FLT3 inhibitor, which was indicated by the aberrant FLT3 signaling in NUP98-NSD1-positive AML patients [80,81]. Besides, AML cases with a co-existing NUP98-NSD1 and FLT3-ITD mutation usually have a poor prognosis, meagre complete remission rates, and a poor chance of survival [80,82]. On account of that, novel treatment strategies are critically required. Kivioja et al. (2019) reported that a combination of Src/Abl-inhibitor dasatinib and BCL-2 inhibitor navitoclax produces a synergistic effect against NUP98-NSD1^+^/FLT3-ITD^+^ AML cells. Patient cells with NUP98-NSD1^+^/FLT3-ITD^+^ were very responsive towards navitoclax and showed the most sensitivity towards dasatinib. Unlike those in healthy CD34+ cells, the expression of the anti-apoptotic BCL-2A1proteins Lck and Fgr were highly upregulated in the NUP98-NSD1^+^/FLT3-ITD^+^ AML cells. They proposed that the high expression of these proteins contributed to sensitizing NUP98-NSD1^+^/FLT3-ITD^+^ AML cells towards navitoclax and dasatinib [83]. Additionally, dasatinib was highly efficacious against those mutated cells indicated by the lowest mean concentration to inhibit a half-maximal response (IC_50_) compared to other tested drugs [83]. Hence, navitoclax combined with dasatinib produces synergistic outcomes against AML cells co-expressing NUP98-NSD1 and FLT3-ITD, in which navitoclax inhibits the BCL-2A1 while dasatinib suppresses the proteins Lck and Fgr [83]. 

#### 3.2.2. Acute Lymphoblastic Leukemia (ALL)

Patients with ALL are often associated with poor prognosis and survival rates due to the effect of the BCR-ABL oncogene. Furthermore, leukemic cells usually express a higher level of anti-apoptotic proteins to prevent cancer cell death. Navitoclax is effective in suppressing the anti-apoptotic BCL-2 and BCL-XL. Similar to the case of pancreatic ductal adenocarcinoma, anti-apoptotic MCL-1 protein levels are also increased in leukemic cells to maintain cancer survival [84]. Based on a study conducted by Budhraja et al. (2017), dihydroartemisinin (DHA), an orally available antimalarial drug, was proven to decrease the expression of MCL-1 in BCR-ABL^+^ mice through the activation of CHOP-dependent cellular stress activity. DHA suppressed the expression of MCL-1 only as the expression levels of both BCL-2 and BCL-XL remain unaffected. Treatment of BCR-ABL^+^ B lineage ALL (B-ALL) cells activated the expression of CHOP, subsequently inducing the endoplasmic reticulum (E.R.) stress pathway and pro-apoptotic Noxa protein. As a result, Noxa triggered the breakdown of the MCL-1 protein through proteasome-mediated degradation [85]. The inhibition of MCL-1 by DHA led to the apoptosis of murine BCR-ABL^+^ B-ALL cells and human leukemic cells. By targeting BCL-XL and MCL-1, a combination of DHA and navitoclax interacted synergistically to kill mouse BCR-ABL^+^ B-ALL cells and suppress the progression of BCR-ABL^+^ B-ALL leukemia in vivo.

**Table 1:** Summary of navitoclax in combination with other drugs.

Disease	Model	Combination Drug	Advantages/Disadvantages	Ref.
Metastatic melanoma	A375 and SKMEL-28 cell lines, and mouse xenograft tumor	S63845 + navitoclax	Induce melanoma cell death by blocking MCL-1 and BCL-2	[58]
Rhabdomyosarcoma (RMS)	RMS cell lines, patient-derived xenograft (PDX) tumors and mouse xenograft tumor	Navitoclax + alisertib	Enhance intrinsic apoptosis of RMS cancer cells after the loss of PAX3-FOXO1 protein in a Noxa-dependent mechanism	[60]
Endometrial carcinoma	Human endometrial adenocarcinoma cell line Ishikawa and mouse xenograft model	Doxorubicin (DOX) + navitoclax	Increase DOX effect and stimulate cancer cell apoptosis by blocking BCL-2	[64]
Papillary thyroid cancer (PTC)	K1 human BRAF^V600E^-positive PTC cell line	Navitoclax + vemurafenib	Induce apoptosis by blocking p-Erk 1/2, BCL-XL and BCL-2	[69]
Pancreatic ductal adenocarcinoma (PDAC)	Pancreatic cell lines and HeLa cell line	Aminopyrazole analogue 24 + navitoclax	Induce apoptosis via concurrent inactivation of MCL-1 (indirectly) and BCL-XL	[76]
Small cell lung carcinoma (SCLC)	H69, H526, H82 and H209 human SCLC cell lines	Vorinostat + navitoclax	Induce Noxa to degrade MCL-1 and inhibit BCL-XL to enhance cancer cell death	[46]
Acute myeloid leukemia (AML)	Bone marrow cells from NUP98-NSD1^+^/FLT3-ITD^+^ and NUP98-NSD1^-^/FLT3-ITD^+^ AML patients	Navitoclax + dasatinib	Increase cell death by suppressing BCL-2A1, proteins Lck and Fgr	[83]
Acute lymphoblastic leukemia (ALL)	Murine BCR-ABL^+^ B-ALL cells and patient-derived xenograft mice	Dihydroartemisinin (DHA) + navitoclax	Downregulate MCL-1 expression and inhibit BCL-XL activity from killing BCR-ABL^+^ B lineage ALL (B-ALL) cells	[85]

## 4. Conclusions and Future Prospects

Overall, BCL-2 family members play an essential role in the regulation of cell apoptosis and survival. The dysregulation of BCL-2 proteins results in cell resistance to apoptosis. However, the induced overexpression of anti-apoptotic BCL-2 proteins in cancer cells can provide a new therapeutic strategy to inhibit cancer cell progression and metastasis. Identifying BCL-2 family protein expressions in different tumor types is fundamentally essential to assist in choosing the relevant BCL-2 inhibitors in a combination treatment. Based on the evidence discussed in this review, navitoclax combination therapy in solid and non-solid tumors has been investigated to treat advanced malignancies that are resistant to a single anti-cancer drug [58,61] or to treat cancer relapsed following treatment with a monotherapy agent [60,69]. Navitoclax is known to be a potent and selective inhibitor of BCL-2 and BCL-XL. The effect of navitoclax is not restricted by cell types as its efficacy is proven in a wide range of cancer cell types. Researchers have shown that the function of navitoclax as a BCL-2 family inhibitor can be ensured when the cells have an elevated expression of BCL-2 proteins. Navitoclax has been widely applied in the combination treatment of various cancer types, such as SCLC, endometrial carcinoma, acute myeloid leukemia, and others. However, the complete mechanism of action is still not fully understood. Besides, the timing of navitoclax administration in specific cancer types should be well studied in the future to optimize the effect of navitoclax in overcoming relapsed cancer. To be aware of any possible adverse side effects, a deeper investigation should be conducted to elucidate the interaction of navitoclax with cellular molecules and its downstream metabolic activities in combination with other chemotherapeutic agents. 

## References

[1] Hanahan D., Weinberg R.A. (2000). The hallmarks of cancer. Cell.

[2] Hanahan D., Weinberg R.A. (2011). Hallmarks of cancer: The next generation. Cell.

[3] Fouad Y.A., Aanei C. (2017). Revisiting the hallmarks of cancer. Am. J. Cancer Res..

[4] Llambi F., Green D.R. (2011). Apoptosis and oncogenesis: Give and take in the BCL-2 family. Curr. Opin. Genet. Dev..

[5] Perez-mancera P.A., Young A.R.J., Narita M. (2014). Inside and out: The activities of senescence in cancer. Nat. Rev. Cancer.

[6] Labi V., Erlacher M. (2015). How cell death shapes cancer. Cell Death Dis..

[7] Razak N.A., Abu N., Ho W.Y., Zamberi N.R., Tan S.W., Alitheen N.B., Long K., Yeap S.K. (2019). Cytotoxicity of eupatorin in MCF-7 and MDA-MB-231 human breast cancer cells via cell cycle arrest, anti-angiogenesis and induction of apoptosis. Sci. Rep..

[8] Zhao C., Yang H., Shi H., Wang X., Chen X., Yuan Y., Lin S., Wei Y. (2011). Distinct contributions of angiogenesis and vascular co-option during the initiation of primary microtumors and micrometastases. Carcinogenesis.

[9] Valastyan S., Weinberg R.A. (2011). Tumor metastasis: Molecular insights and evolving paradigms. Cell.

[10] Pavlova N.N., Thompson C.B. (2016). The emerging hallmarks of cancer metabolism. Cell Metab..

[11] DeBeradinis R.J., Chandel N.S. (2016). Fundamentals of cancer metabolism. Sci. Adv..

[12] Palanissami G., Paul S.F.D. (2018). RAGE and its ligands: Molecular interplay between glycation, inflammation, and halmarks of cancer—A Review. Horm. Cancer.

[13] Czabotar P.E., Lessene G., Strasser A., Adams J.M. (2014). Control of apoptosis by the BCL-2 protein family: Implications for physiology and therapy. Nat. Rev. Mol. Cell Biol..

[14] Reed J.C. (2003). Apoptosis-targeted therapies for cancer. Cancer Cell.

[15] Amundson S.A., Myers T.G., Scudiero D., Kitada S., Reed J.C., Fornace A.J. (2000). An informatics approach identifying markers of chemosensitivity in human cancer cell lines. Cancer Res..

[16] D’Aguanno S., Del Bufalo D. (2020). Inhibition of anti-apoptotic Bcl-2 proteins in preclinical and clinical studies: Current overview in cancer. Cells.

[17] Campbell K.J., Tait S.W.G. (2018). Targeting BCL-2 regulated apoptosis in cancer. Open Biol..

[18] Tan J.K., Then S.M., Mazlan M., Raja Abdul Rahman R.N., Jamal R., Wan Ngah W.Z. (2016). Gamma-tocotrienol acts as a BH3 mimetic to induce apoptosis in neuroblastoma SH-SY5Y cells. J. Nutr. Biochem..

[19] Ng S.Y., Davids M.S. (2014). Selective Bcl-2 inhibition to treat chronic lymphocytic leukemia and non-hodgkin lymphoma. Clin. Adv. Hematol. Oncol..

[20] ClinicalTrials.gov. https://clinicaltrials.gov/ct2/results?cond=&term=bcl-2+inhibitor%2C+cancer&cntry=&state=&city=&dist=&Search=Search.

[21] Raedler L.A. (2017). Venclexta (Venetoclax) first BCL-2 inhibitor approved for high-risk relapsed chronic lymphocytic leukemia. J. Hematol. Oncol. Pharm..

[22] Reed J.C. (2018). Bcl-2 on the brink of breakthroughs in cancer treatment. Cell Death Differ..

[23] Tse C., Shoemaker A.R., Adickes J., Anderson M.G., Chen J., Jin S., Johnson E.F., Marsh K.C., Mitten M.J., Nimmer P. (2008). ABT-263: A potent and orally bioavailable Bcl-2 family inhibitor. Cancer Res..

[24] Delbridge A.R.D., Strasser A. (2015). The BCL-2 protein family, BH3-mimetics and cancer therapy. Cell Death Differ..

[25] Roberts A.W., Seymour J.F., Brown J.R., Wierda W.G., Kipps T.J., Khaw S.L., Carney D.A., He S.Z., Huang D.C.S., Xiong H. (2012). Substantial susceptibility of chronic lymphocytic leukemia to BCL2 inhibition: Results of a phase I study of Navitoclax in patients with relapsed or refractory disease. J. Clin. Oncol..

[26] Mohamad Anuar N.N., Nor Hisam N.S., Liew S.L., Ugusman A. (2020). Clinical review: Navitoclax as a pro-apoptotic and anti-fibrotic agent. Front. Pharmacol..

[27] Tahir S.K., Wass J., Joseph M.K., Devanarayan V., Hessler P., Zhang H., Elmore S.W., Kroeger P.E., Tse C., Rosenberg S.H. (2010). Identification of expression signatures predictive of sensitivity to the Bcl-2 family member inhibitor ABT-263 in small cell lung carcinoma and leukemia/lymphoma cell Lines. Mol. Cancer Ther..

[28] Ackler S., Mitten M.J., Foster K., Oleksijew A., Refici M., Tahir S.K., Xiao Y., Tse C., Frost D.J., Fesik S.W. (2010). The Bcl-2 Inhibitor ABT-263 enhances the response of multiple chemotherapeutic regimens in hematologic tumors In Vivo. Cancer Chemother. Pharmacol..

[29] Falzone L., Salomone S., Libra M. (2018). Evolution of cancer pharmacological treatments at the turn of the third millennium. Front. Pharmacol..

[30] DeVita V.T., DeVita-Raeburn E., Moxley J.H. (2016). Intensive combination chemotherapy and X-irradiation in Hodgkin’s disease. Cancer Res..

[31] Moxley J.H., De Vita V.T., Brace K., Frei E. (1967). Intensive combination chemotherapy and X-irradiation in Hodgkin’s disease. Cancer Res..

[32] Devita V.T., Serpick A.A., Carbone P.P. (1970). Combination chemotherapy in the treatment of advanced Hodgkin’s disease. Ann. Intern. Med..

[33] Peng P.J., Cheng H., Ou X.Q., Zeng L.J., Wu X., Liu Y.M., Lin Z., Tang Y.N., Wang S.Y., Zhang H.Y. (2014). Safety and efficacy of S-1 chemotherapy in recurrent and metastatic nasopharyngeal carcinoma patients after failure of platinum-based chemotherapy: Multi-institutional retrospective analysis. Drug Des. Dev. Ther..

[34] Ershler W.B. (2006). Capecitabine monotherapy: Safe and effective treatment for metastatic breast cancer. Oncologist.

[35] Vogel C.L., Nabholtz J. (1999). Monotherapy of metastatic breast cancer: A review of newer agents. Oncologist.

[36] Suvarna V., Singh V., Murahari M. (2019). Current Overview on the Clinical Update of Bcl-2 anti-apoptotic inhibitors for cancer therapy. Eur. J. Pharmacol..

[37] Stevens M., Frobisher C., Hawkins M., Jenney M., Lancashire E., Reulen R., Taylor A., Winter D. (2008). The British childhood cancer survivor study: Objectives, methods, population structure, response rates and initial descriptive information. Pediatr. Blood Cancer.

[38] Kim K.B., Cabanillas M.E., Lazar A.J., Williams M.D., Sanders D.L., Ilagan J.L., Nolop K., Lee R.J., Sherman S.I. (2013). Clinical responses to Vemurafenib in patients with metastatic papillary thyroid cancer harboring BRAFV600E mutation. Thyroid.

[39] Johnson D.B., Estrada M.V., Salgado R., Sanchez V., Doxie D.B., Opalenik S.R., Vilgelm A.E., Feld E., Johnson A.S., Greenplate A.R. (2016). Melanoma-specific MHC-II expression represents a tumour-autonomous phenotype and predicts response to anti-PD-1/PD-L1 therapy. Nat. Commun..

[40] Shen Q., Li J., Mai J., Zhang Z., Fisher A., Wu X., Li Z., Ramirez M.R., Chen S., Shen H. (2018). Sensitizing non-small cell lung cancer to BCL-XL-targeted apoptosis. Cell Death Dis..

[41] William W.N., Glisson B.S. (2011). Novel strategies for the treatment of small-cell lung carcinoma. Nat. Rev. Clin. Oncol..

[42] Yap T.A., Omlin A., De Bono J.S. (2013). Development of therapeutic combinations targeting major cancer signaling pathways. J. Clin. Oncol..

[43] Bayat Mokhtari R., Homayouni T.S., Baluch N., Morgatskaya E., Kumar S., Das B., Yeger H. (2017). Combination therapy in combating cancer. Oncotarget.

[44] Schirrmacher V. (2019). From chemotherapy to biological therapy: A review of novel concepts to reduce the side effects of systemic cancer treatment (review). Int. J. Oncol..

[45] Baudino T.A. (2015). Targeted cancer therapy: The next generation of cancer treatment. Curr. Drug Discov. Technol..

[46] Nakajima W., Sharma K., Hicks M.A., Le N., Brown R., Krystal G.W., Harada H. (2016). Combination with Vorinostat overcomes ABT-263 (Navitoclax) resistance of small cell lung cancer. Cancer Biol. Ther..

[47] Anighoro A., Bajorath J., Rastelli G. (2014). Polypharmacology: Challenges and opportunities in drug discovery department of life science informatics, B-IT, LIMES program unit chemical biology and medicinal. J. Med. Chem..

[48] Liu Q., Wang H.G. (2012). Anti-cancer drug discovery and development Bcl-2 family small molecule inhibitors. Commun. Integr. Biol..

[49] Ibrahim N., Nazimi A.J., Ajura A.J., Nordin R., Latiff Z.A., Ramli R. (2016). The clinical features and expression of bcl-2, Cyclin D1, p53, and proliferating cell nuclear antigen in syndromic and nonsyndromic keratocystic odontogenic tumor. J. Craniofac. Surg..

[50] Wolter K.G., Wang S.J., Henson B.S., Wang S., Griffith K.A., Kumar B., Chen J., Carey T.E., Bradford C.R., D’Silva N.J. (2006). (-)-Gossypol inhibits growth and promotes apoptosis of human head and neck squamous cell carcinoma In Vivo. Neoplasia.

[51] Schwab E., Chen J., Huynh J., Ji J., Arora M., Cho M., Kim E. (2019). Rational strategies for combining Bcl-2 inhibition with targeted drugs for anti-tumor synergy. J. Cancer Treat. Diagn..

[52] Shoemaker A.R., Mitten M.J., Adickes J., Ackler S., Refici M., Ferguson D., Oleksijew A., O’Connor J.M., Wang B., Frost D.J. (2008). Activity of the Bcl-2 family inhibitor ABT-263 in a panel of small cell lung cancer xenograft models. Clin. Cancer Res..

[53] Shi J., Zhou Y., Huang H.-C., Mitchison T.J. (2011). Navitoclax (ABT-263) accelerates apoptosis during drug-induced mitotic arrest by antagonizing Bcl-XL. Cancer Res..

[54] Lee E.Y., Gong E.Y., Shin J.S., Moon J.H., Shim H.J., Kim S.M., Lee S., Jeong J., Gong J.H., Kim M.J. (2018). Human breast cancer cells display different sensitivities to ABT-263 based on the level of survivin. Toxicol. Vitr..

[55] Yang I.H., Jung J.Y., Kim S.H., Yoo E.S., Cho N.P., Lee H., Lee J.Y., Hong S.D., Shin J.A., Cho S.D. (2019). ABT-263 exhibits apoptosis-inducing potential in oral cancer cells by targeting C/EBP-homologous protein. Cell. Oncol..

[56] Gandhi L., Camidge D.R., De Oliveira M.R., Bonomi P., Gandara D., Khaira D., Hann C.L., McKeegan E.M., Litvinovich E., Hemken P.M. (2011). Phase I study of Navitoclax (ABT-263), a novel Bcl-2 family inhibitor, in patients with small-cell lung cancer and other solid tumors. J. Clin. Oncol..

[57] Rudin C.M., Hann C.L., Garon E.B., Ribeiro De Oliveira M., Bonomi P.D., Camidge D.R., Chu Q., Giaccone G., Khaira D., Ramalingam S.S. (2012). Phase II study of single-agent Navitoclax (ABT-263) and biomarker correlates in patients with relapsed small cell lung cancer. Clin. Cancer Res..

[58] Mukherjee N., Skees J., Todd K.J., West D.A., Lambert K.A., Robinson W.A., Amato C.M., Couts K.L., Van Gulick R., Macbeth M. (2020). MCL1 inhibitors S63845/MIK665 plus Navitoclax synergistically kill difficult-to-treat melanoma cells. Cell Death Dis..

[59] Missiaglia E., Williamson D., Chisholm J., Wirapati P., Pierron G., Petel F., Concordet J.-P., Thway K., Oberlin O., Pritchard-Jones K. (2012). PAX3/FOXO1 fusion gene status is the key prognostic molecular marker in Rhabdomyosarcoma and significantly improves current risk stratification. J. Clin. Oncol..

[60] Ommer J., Selfe J.L., Wachtel M., O’Brien E.M., Laubscher D., Roemmele M., Kasper S., Delattre O., Surdez D., Petts G. (2020). Aurora A kinase inhibition destabilizes PAX3-FOXO1 and MYCN and synergizes with Navitoclax to induce Rhabdomyosarcoma cell death. Cancer Res..

[61] Gibson W.J., Hoivik E.A., Halle M.K., Taylor-Weiner A., Cherniack A.D., Berg A., Holst F., Zack T.I., Werner H.M.J., Staby K.M. (2016). The genomic landscape and evolution of endometrial carcinoma progression and abdominopelvic metastasis. Nat. Genet..

[62] Palisoul M., Mutch D.G. (2016). The clinical management of inoperable endometrial carcinoma. Expert Rev. Anticancer Ther..

[63] Jurado R., Lopez-flores A., Alvarez A., García-López P. (2009). Cisplatin cytotoxicity is increased by Mifepristone in cervical carcinoma: An In Vitro and In Vivo study. Oncol. Rep..

[64] Ding J., Zhang X., Chen C., Huang Y., Yu X., Li X. (2020). Ultra PH-sensitive polymeric nanovesicles co-deliver Doxorubicin and Navitoclax for synergetic therapy of endometrial carcinoma. Biomater. Sci..

[65] Zhu G., Deng Y., Pan L., Ouyang W., Feng H., Wu J., Chen P., Wang J., Chen Y., Luo J. (2018). Clinical significance of the BRAF V600E mutation in PTC and its effect on radioiodine therapy. Endocr. Connect..

[66] McArthur G.A., Chapman P.B., Robert C., Larkin J., Haanen J.B. (2014). Safety and efficacy of Vemurafenib in BRAFV600E and BRAFV600K mutation-positive melanoma (BRIM-3): Extended follow-up of a phase 3, randomised, open-label study. Lancet Oncol..

[67] Brose M.S., Cabanillas M.E., Cohen E.E.W., Wirth L.J., Riehl T., Yue H., Sherman P.S.I., Sherman E.J. (2017). Vemurafenib in patients with BRAFV600E-positive metastatic or unresectable papillary thyroid cancer refractory to radioactive iodine: A non-randomized, multicentre, open-label, phase 2 trial. Lancet Oncol..

[68] Dadu R., Shah K., Busaidy N.L., Waguespack S.G., Habra M.A., Ying A.K., Hu M.I., Bassett R., Jimenez C., Sherman S.I. (2014). Efficacy and tolerability of Vemurafenib in patients with BRAFV600E-positive papillary thyroid cancer: M.D. Anderson cancer center off label experience. J. Clin. Endocrinol. Metab..

[69] Jeong J.H., Oh J.M., Jeong S.Y., Lee S.W., Lee J., Ahn B.C. (2019). Combination treatment with the BRAF V600E inhibitor Vemurafenib and the BH3 mimetic Navitoclax for BRAF-mutant thyroid carcinoma. Thyroid.

[70] Siegel R.L., Miller K.D., Jemal A. (2016). Cancer statistics, 2016. CA Cancer J. Clin..

[71] Abulwerdi F., Liao C., Liu M., Azmi A.S., Aboukameel A., Mady A.S., Gulappa T., Cierpicki T., Owens S., Zhang T. (2015). A novel small-molecule inhibitor of Mcl-1 blocks pancreatic cancer growth In Vitro and In Vivo. Mol. Cancer Ther..

[72] Takahashi H., Chen M.C., Pham H., Matsuo Y., Ishiguro H., Reber H.A., Takeyama H., Hines O.J., Eibl G. (2013). Simultaneous knock-down of Bcl-XL and Mcl-1 induces apoptosis through Bax activation in pancreatic cancer cells. Biochim. Biophys. Acta BBA.

[73] Mazumder S., Choudhary G.S., Al-harbi S., Almasan A. (2012). Mcl-1 Phosphorylation defines ABT-737 resistance that can be overcome by increased NOXA expression in leukemic B-cells. Cancer Res..

[74] Abid M., Sonawane Y.A., Contreras J.I., Rana S., Natarajan A. (2017). Recent advances in cancer drug development: Targeting induced myeloid cell leukemia-1 (Mcl-1) differentiation protein. Curr. Med. Chem..

[75] Lowman X.H., Mcdonnell M.A., Kosloske A., Odumade O.A., Jenness C., Karim C.B., Jemmerson R., Kelekar A. (2010). The pro-apoptotic function of Noxa in human leukemia cells is regulated by the kinase Cdk5 and by glucose. Mol. Cell.

[76] Kour S., Rana S., Contreras J.I., King H.M., Robb C.M., Sonawane Y.A., Bendjennat M., Crawford A.J., Barger C.J., Kizhake S. (2019). CDK5 inhibitor downregulates Mcl-1 and sensitizes pancreatic cancer cell lines to Navitoclax. Mol. Pharmacol..

[77] Surien O., Ghazali A.R., Masre S.F. (2019). Lung cancers and the roles of natural compounds as potential chemotherapeutic and chemopreventive agents. Biomed. Pharmacol. J..

[78] Inoue S., Riley J., Gant T.W., Dyer M.J.S., Cohen G.M. (2007). Apoptosis induced by histone deacetylase inhibitors in leukemic cells is mediated by Bim and Noxa. Leukemia.

[79] Hollink I.H.I.M., Van Den Heuvel-Eibrink M.M., Arentsen-Peters S.T.C.J.M., Pratcorona M., Abbas S., Kuipers J.E., Van Galen J.F., Beverloo H.B., Sonneveld E., Kaspers G.J.J.L. (2011). NUP98/NSD1 characterizes a novel poor prognostic group in acute myeloid leukemia with a distinct HOX gene expression pattern. Blood J. Am. Soc. Hematol..

[80] Thanasopoulou A., Tzankov A., Schwaller J. (2014). Potent co-operation between the NUP98-NSD1 fusion and the FLT3-ITD mutation in acute myeloid leukemia induction. Haematologica.

[81] Akiki S., Dyer S.A., Grimwade D., Ivey A., Abou-zeid N., Borrow J., Jeffries S., Caddick J., Newell H., Begum S. (2013). NUP98-NSD1 fusion in association with FLT3-ITD mutation identifies a prognostically relevant subgroup of pediatric acute myeloid leukemia patients suitable for monitoring by real time quantitative PCR. Genes Chromosomes Cancer.

[82] Ostronoff F., Othus M., Gerbing R.B., Loken M.R., Raimondi S.C., Hirsch B.A., Lange B.J., Petersdorf S., Radich J., Appelbaum F.R. (2014). NUP98/NSD1 and FLT3/ITD coexpression is more prevalent in younger aml patients and leads to induction failure: A COG and SWOG report. Blood J. Am. Soc. Hematol..

[83] Kivioja J.L., Thanasopoulou A., Kumar A., Kontro M., Yadav B., Majumder M.M., Javarappa K.K., Eldfors S., Schwaller J., Porkka K. (2019). Dasatinib and Navitoclax act synergistically to target NUP98-NSD1^+^/FLT3-ITD^+^ acute myeloid leukemia. Leukemia.

[84] Koss B., Morrison J., Perciavalle R.M., Singh H., Rehg J.E., Williams R.T., Opferman J.T. (2013). Requirement for antiapoptotic MCL-1 in the survival of BCR-ABL B-lineage acute lymphoblastic leukemia. Blood J. Am. Soc. Hematol..

[85] Budhraja A., Turnis M.E., Churchman M.L., Kothari A., Yang X., Xu H., Kaminska E., Panetta J.C., Finkelstein D., Mullighan C.G. (2017). Modulation of Navitoclax sensitivity by Dihydroartemisinin-mediated MCL-1 repression in BCR-ABL^+^ B-lineage acute lymphoblastic leukemia. Clin. Cancer Res..

